# An All-Payer Risk Model for Super-Utilization in a Large Safety Net System

**DOI:** 10.1007/s11606-018-4346-5

**Published:** 2018-02-20

**Authors:** Jeremy Ziring, Spriha Gogia, Remle Newton-Dame, Jesse Singer, Dave A. Chokshi

**Affiliations:** 1 NYU School of Medicine, New York, NY USA; 2 NYC Health + Hospitals, New York, NY USA

## INTRODUCTION

Identifying patients at high risk for super-utilization of inpatient and emergency services—and proactively managing their care—are key strategies for healthcare systems aiming to improve population health and control costs. Traditional claims-based risk scores are inadequate for uninsured patients and patients with insurance churn, and many safety net systems do not have an electronic health record (EHR) capable of advanced analytics.

As the largest safety net system in the country, NYC Health + Hospitals serves a high-need population, including thousands of patients with multiple, interlinked medical, behavioral health, and social issues. More than half of the system’s patients had an emergency room (ER) visit in the past year. Seventeen percent had two or more visits, and 250 patients averaged at least a day a week in one of our emergency rooms. NYC Health + Hospitals also provides half of all uninsured emergency and inpatient care for New Yorkers, including more than 80% of uninsured non-emergency services.^1^ To be successful, risk prediction strategies must encompass NYC Health + Hospitals’ entire patient population.

## METHODS

Our objective was to develop a payer-agnostic risk model for super-utilization using administrative and clinical data from the largest safety net system in the USA. We selected adults that visited an NYC Health + Hospitals acute care, community health center, or skilled nursing facility in 2014 (index year) and were not designated pregnant or actively incarcerated during the study period. Patients were randomly assigned to a development (80%) or validation (20%) cohort, using SAS Enterprise Guide 7.11 (SAS Institute). Internal administrative data provided utilization, demographic and scheduling data, and diagnoses came from clinical data. Our primary outcome was super-utilization within our system during 2015 (prediction year), defined as ≥ 10 days in inpatient care or the emergency room. To identify candidate variables, we aligned available data elements to key factors in the literature and clinical guidelines.^2^ Stepwise selection identified the final logistic model and generated algorithmic weights; model discrimination was assessed using the c-statistic. Our large sample size precluded the Hosmer-Lemeshow test.^3^ We converted weights to a risk algorithm using methods described by Sullivan et al.^4^ Risk score performance was assessed on the validation set using positive predictive value for 2015 super-utilization among the top 1% of 2014 patients (high risk). Figure was generated using Tableau 10.2 (Tableau Software).

## RESULTS

We retained 643,475 NYC Health + Hospitals patients in the development cohort, of which 45.8% were males, 33.9% were enrolled in Medicaid at their most recent visit in 2014, and 37.4% were uninsured. Median age was 45. In 2014, 4.8% of this cohort was super-utilizers, dropping to 2.9% in 2015.

In the final algorithm, the strongest predictors of super-utilization were inpatient and ER visits; older age; and diagnoses of schizophrenia, chronic kidney disease, and sickle cell disease (Table 1). We did not retain race, which was significant, due to data validity concerns. The final model’s c-statistic was 0.86, outperforming established readmission models tested at other urban public hospitals and falling above the 0.70 threshold of acceptable discrimination.^5^

**Table 1:** Payer-Agnostic Risk Score from a 2014/2015 Safety Net Cohort

Variable	Value	Risk points
ED visits in 2014	1–2 visits	2.4
	3–4 visits	5.7
	5+ visits	10.3
Inpatient visits in 2014	1 visit	6.7
	2 visits	8.2
	3 visits	9.1
	4+ visits	11.3
≥ 10 inpatient/ED days in 2014		3.9
Marital status category	Single, never married	1.9
	Separated, widowed, and divorced	3.1
Gender	Male	1.7
Age category	45–64	2.6
	65–80	6.1
	≥ 81	9.4
Diagnosis flags	Alcohol disorders	4.2
	Schizophrenia	7.7
	Mood disorders	1.7
	Heart disease	1.4
	Substance disorders	2.6
	Chronic kidney disease	5.6
	Asthma	1.7
	Diabetes	1.1
	Sickle cell	5.6
≥ 2 missed outpatient medicine clinic visits		1.0
Zip changes	1–2 zip changes	1.8
	3+ zip changes	2.3
Payer changes	1–2 payer changes	1.0
	3–4 payer changes	1.4
	5+ payer changes	2.0

**Fig. 1:**
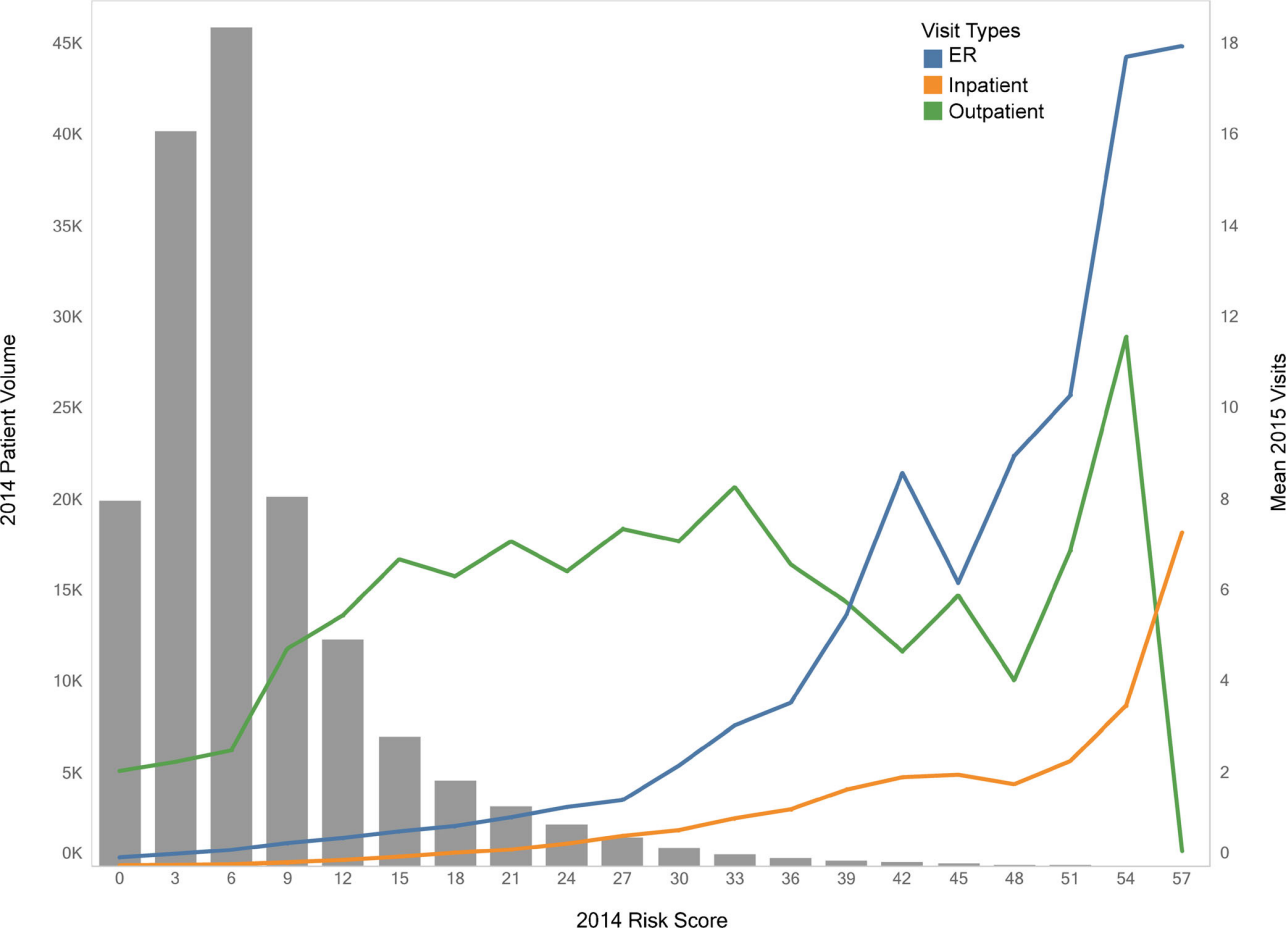
Mean 2015 emergency, inpatient, and outpatient utilization vs 2014 risk score distribution among NYC Health + Hospitals patients.

In the validation set (*n* = 160,868), the model identified 2015 super-utilization among 2014 high risk patients with a 44.8% positive predictive value. In 2015, high-risk patients had an average of 1.5 inpatient and 5.4 ER visits, and 75.5% had ≥ 1 ER/inpatient visit. As risk of super-utilization increased, average ER/inpatient visits rose while outpatient visits leveled off and then declined (Fig. 1).

## DISCUSSION

Readily available clinical and administrative data successfully discriminated risk of future super-utilization for both uninsured and insured patients across all payers at a large, urban safety net hospital system. Proxies such as zip code changes, payer flux, and missed clinic visits helped represent poorly documented social determinants of health. This model did not require advanced EHR functionality or proprietary claim-based rules, making it timely and affordable for our system. A payer-agnostic approach to risk scoring may increase clinician buy-in, since it covers the provider’s full panel of patients and improves targeting of resource-intensive interventions. We hope that this algorithm helps initiate thoughtful, population-targeted risk stratification strategies at other delivery systems serving vulnerable patients.
